# A microfluidic device for isolation and characterization of transendothelial migrating cancer cells

**DOI:** 10.1063/1.4974012

**Published:** 2017-01-13

**Authors:** Xin Cui, Weijin Guo, Yubing Sun, Baoce Sun, Shuhuan Hu, Dong Sun, Raymond H. W. Lam

**Affiliations:** 1 Department of Mechanical and Biomedical Engineering, City University of Hong Kong, Kowloon, Hong Kong; 2 Department of Mechanical and Industrial Engineering, University of Massachusetts Amherst, Amherst, Massachusetts 01002, USA; 3 Centre for Robotics and Automation, City University of Hong Kong, Kowloon, Hong Kong; 4 Centre for Biosystems, Neuroscience, and Nanotechnology, City University of Hong Kong, Kowloon, Hong Kong; 5 City University of Hong Kong Shenzhen Research Institute, Shenzhen, China

## Abstract

Transendothelial migration of cancer cells is a critical stage in cancer, including breast cancer, as the migrating cells are generally believed to be highly metastatic. However, it is still challenging for many existing platforms to achieve a fully covering endothelium and to ensure transendothelial migration capability of the extracted cancer cells for analyses with high specificity. Here, we report a microfluidic device containing multiple independent cell collection microchambers underneath an embedded endothelium such that the transendothelial-migrated cells can be selectively collected from only the microchambers with full coverage of an endothelial layer. In this work, we first optimize the pore size of a microfabricated supporting membrane for the endothelium formation. We quantify transendothelial migration rates of a malignant human breast cell type (MDA-MB-231) under different shear stress levels. We investigate characteristics of the migrating cells including morphology, cytoskeletal structures, and migration (speed and persistence). Further implementation of this endothelium-embedded microfluidic device can provide important insights into migration and intracellular characteristics related to cancer metastasis and strategies for effective cancer therapy.

## INTRODUCTION

Breast cancer is well known as the second leading cause of cancer-related deaths among women.^1^ Remarkably, its metastasis, rather than the primary tumors, causes most of the deaths.^2^ In the metastasis, tumor cells escaped to the bloodstream can further extravasate to distant tissues or organs through membranes of the lymphatic and hematogenous systems.^3^ Understanding behaviors of the cancer cells migrated through vascular endothelial layers is essential to reveal fundamental mechanisms of metastasis as well as the related cancer therapeutics.

A variety of functional metastasis assays have been developed to quantify the adhesion, migration, invasion, and proliferation of tumor cells in response to various stimuli.^4^ Transwells or modified Boyden chamber assays have been widely used to study the cancer cell transendothelial migration.^5^ Vascular endothelial cells can be pre-placed on a porous membrane and grow as an endothelium before seeding cancer cells.^6^ This approach allows researchers to monitor migration of cancer cells across the endothelium under a chemotactic gradient. However, it is still challenging to control fluidic conditions to mimic the bloodstream together with specific biochemical conditions such as a defined chemotactic gradient over time.

Recent advances in microfluidics enable sorting^7^ and comprehensive analyses^8–11^ of cells in a reduced biopsy quantity under more precise controls on the shear stress and chemical gradients.^12–15^ Many microfluidic devices have been developed for cancer metastasis research.^16,17^ For instance, Swaminathan *et al.* reported a multi-step microfluidic device capable of tracking individual breast cancer cells invading through matrigel-coated microgaps lined with human microvascular endothelial cells.^18^ Kamm *et al.* developed a three-dimensional microfluidic model for live-cell imaging of tumor cell intravasation into collagen hydrogel. They also investigated the roles of inflammatory factors present in the tumor microenvironment.^19,20^ On the other hand, microstructured porous sidewalls with well-defined dimensions were utilized as the membrane for transendothelial migration^6,21^ and angiogenesis analyses,^22^ yet design inflexibilities such as the sidewall porosity and the through-hole size and shape remained limitations of these *in vitro* platforms. Microfluidics has also been applied to characterize cancer cell migration via extracellular matrices with a three-dimensional configuration.^23,24^

Metastatic cells involve a broad spectrum of migration processes, including amoeboid and chain motility.^25^ Phenotypic, genetic, and epigenetic states of cancer cells are well known to be related to their transendothelial migration and metastatic potentials with high specificity.^26–29^ Although previous metastatic platforms have demonstrated live-cell imaging and characterization of cancer cells during the migration process,^30,31^ it is important to distinguish whether the migrated cells are those migrated through an endothelium or majorly the ones flowing through pores on the microstructured membrane. Nonetheless, very few of the reported endothelium-embedded platforms can ensure full coverage of the endothelium without adding promoting molecules such as zonula occludens-1 and endothelial-cadherin.^32^ It remains difficult to apply the existing platforms to specifically isolate only the cancer cells after migrating through an endothelium, to perform more detailed mechanistic study on metastasis, and to develop new therapeutic approaches targeting those transendothelial-migrating cancer cells.

In this work, we present a microfluidic transendothelial migration assay integrated with a biocompatible porous membrane and an array of independently controlled microchambers for selecting the cells migrated through an endothelium. Instead of ensuring the fully covering endothelial, the device design enables selection of cell extraction only for the sub-regions with full coverage of endothelial cells for improving the cell selection selectivity over the conventional Transwells assays. Breast cells are then seeded on the endothelium, migrate through the endothelium under a defined shear stress, and are selectively collected only the cells migrated through a fully covering endothelial layer for further analyses. As demonstration, we utilize this device to examine the transendothelial migration capability of metastatic breast cancer cells, compared with normal breast cells. We also characterize the migration ability and the formation of cytoskeletal components of the migrating cells.

## RESULTS AND DISCUSSION

### Device design

**FIG. 1.**
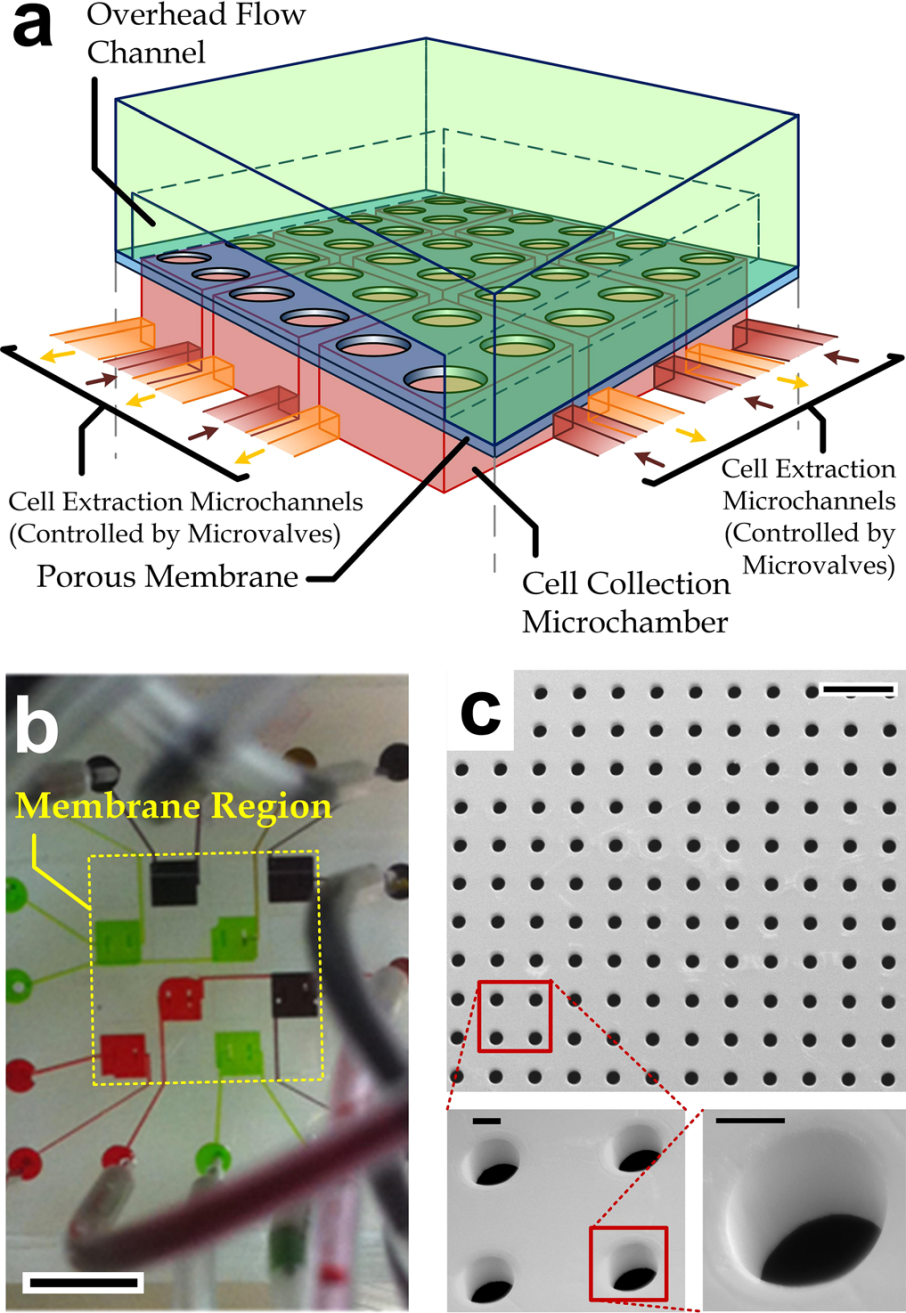
(a) Conceptual design of a microfluidic device for the transendothelial migration study. (b) Photograph of a fabricated device selectively filled with color dye solutions along the cell collection chambers (8 out of the total 16 chambers) underneath the porous membrane. Scale bar: 2 mm. (c) SEM images of a microfabricated SU-8 porous membrane. This membrane contains an array of pores with a common diameter of 20 *μ*m. Scale bar in the top view: 100 *μ*m. Scale bars in insets: 10 *μ*m.

We have developed a multilayer microfluidic extravasation device (Figs. 1(a) and 1(b)) consisting of a microfabricated porous membrane (area: 4 mm × 4 mm), on which primary human vascular endothelial cells can form as an endothelial layer. This device contains two flow layers sandwiching the porous membrane (Fig. 1(c)), which is fabricated by photolithography of the transparent, biocompatible SU-8 photoresist (Young's modulus: ∼20 GPa (Ref. 33)); while the underneath layer contains multiple individually accessible microchamber for cell collection.

The membrane acts as a physical support for the endothelium formation. Compared with conventional track-etched polycarbonate filters and parylene-based micro-pore membranes, pore size and distribution of the SU-8 membrane can be precisely controlled by the microfabrication parameters. The membrane should be pre-coated with an extracellular matrix protein (e.g., fibronectin) for cell adhesion, spreading, and growth. The overhead microchannel allows cell seeding and precise controls of flow conditions (e.g., shear stress) overhead the membrane. An endothelium will form on the membrane if we seed vascular endothelial cells in the device. Subsequently, we can seed another cell type (breast cells or breast cancer cells) on the endothelium for monitoring and analyzing their transendothelial migration properties.

As mentioned, it is technically challenging to ensure full cell coverage over the entire membrane region, and therefore, some cells could pass through the uncovered membrane pores without migrating through the endothelial layer. The cells migrated underneath the membrane may not have the transendothelial migration capabilities. Instead of optimizing culture conditions for achieving the full cell coverage to solve such problem, we adopt an alternative approach of dividing the underneath collection layer into an array of multiple (4 × 4) microchambers (area of each region: 1 mm × 1 mm). Each microchamber contained an independent pair of inlet and outlet channels controlled by pneumatic microvalves.^34^ We have tested the independent accessibility of each microchamber by flowing color dyes along selected microchambers as shown in Fig. 1(b). In this case, the sub-regions can be examined for the full endothelial coverage before they are taken into account for the cell collection. The migrating cells in the selected microchambers are then trypsinized and extracted, followed by subsequent detailed characteristics using the conventional cell analysis techniques.

### Selection of membrane porosity

**FIG. 2.**
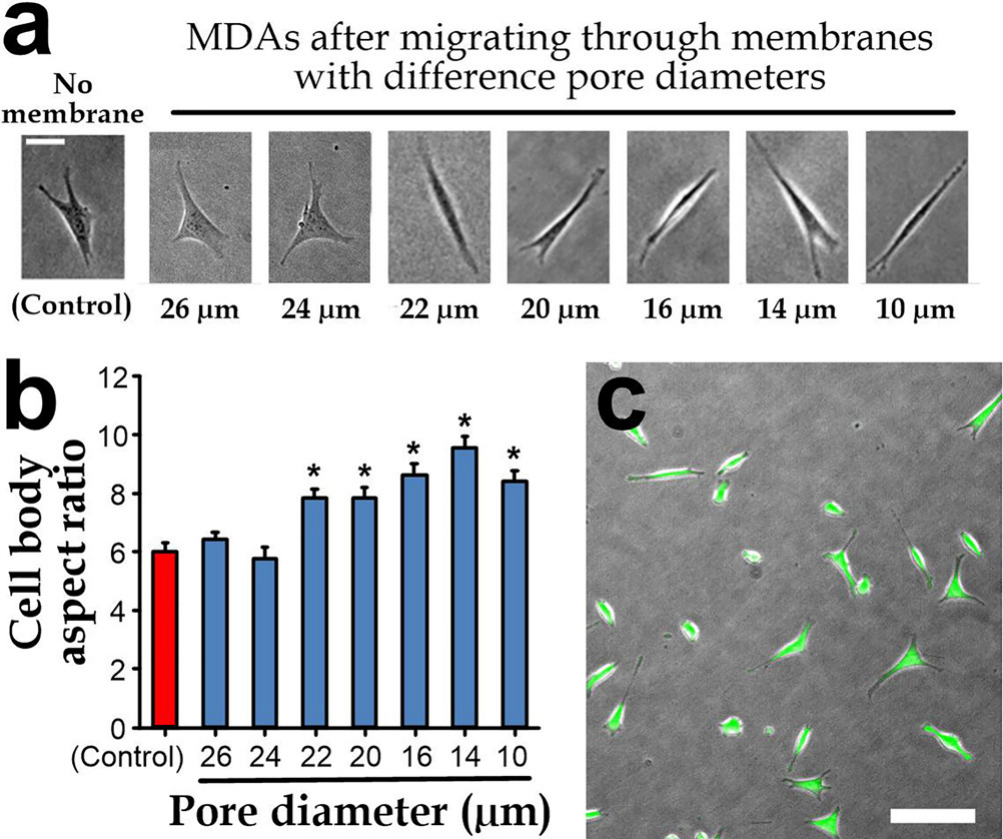
(a) Micrographs of single MDA-MB-231 cells spreading in the collection chambers after migrating through the membranes with different pore sizes. Scale bar: 20 *μ*m. (b) Statistics of aspect ratios of the cell body. (c) Cell viability of MDA-MB-231 cells migrated via a membrane with a pore size of 24 *μ*m. *Green* represents viable cells and *red* represents dead cells. Scale bar: 100 *μ*m.

The porous membrane provides a physical support for formation of the endothelial cell layer; meanwhile, the membrane pores should be large enough without inducing neither pre-selection nor alternation of the cell properties by the physical pore constriction as in most of the conventional Transwells assays, which contain pore sizes smaller than the cells. Accordingly to our preliminary tests (data not shown), a sufficiently small pore diameter (<28 *μ*m) should be designed such that cells can fill pore areas of the membrane by spreading across the pores for formation of an endothelial layer. To further optimize the pore size, we have performed experiments on the membrane-embedded devices with different pore diameters: 10 *μ*m, 14 *μ*m, 16 *μ*m, 18 *μ*m, 20 *μ*m, 22 *μ*m, 24 *μ*m, and 26 *μ*m. Here, we skipped seeding of vascular endothelial cells. We directly seeded breast cancer cells (MDA-MB-231) on the membrane, followed by applying the chemokine CXCL12 (C-X-C Motif Chemokine Ligand 12; concentration: 100 ng/ml) along the collection microchambers below to stimulate cell migration.^35^ It has been well studied that the binding of CXCL12 to C-X-C chemokine receptor type 4 expressed on the breast cancer cell surface can promote the cell invasion and regulate metastasis of breast cancer.^36–38^ After 15 h (Refs. 39 and 40) of cell incubated in the device, we observed morphology of the spreading cells migrated into the collection chambers using a bright-field microscope (Fig. 2(a)). We quantified cell shapes as aspect ratios of their spreading areas, and we found that a pore diameter of ≥24 *μ*m caused negligible effects while cells migrated through membranes with a pore diameter ≤22 *μ*m had a significantly higher aspect ratio (Fig. 2(b)). We further performed the cell viability test on the MDA-MB-231 cells migrated through the pores with a diameter of 24 *μ*m, and the results indicate a perfect viability (98.7 ± SEM0.5% as shown in Fig. 2(c)). Therefore, we have chosen a pore diameter of 24 *μ*m in the reported microfluidic device to eliminate the cell pre-filtering by the physical membrane structure.

### Counting transendothelial migrating breast cancer cells

**FIG. 3.**
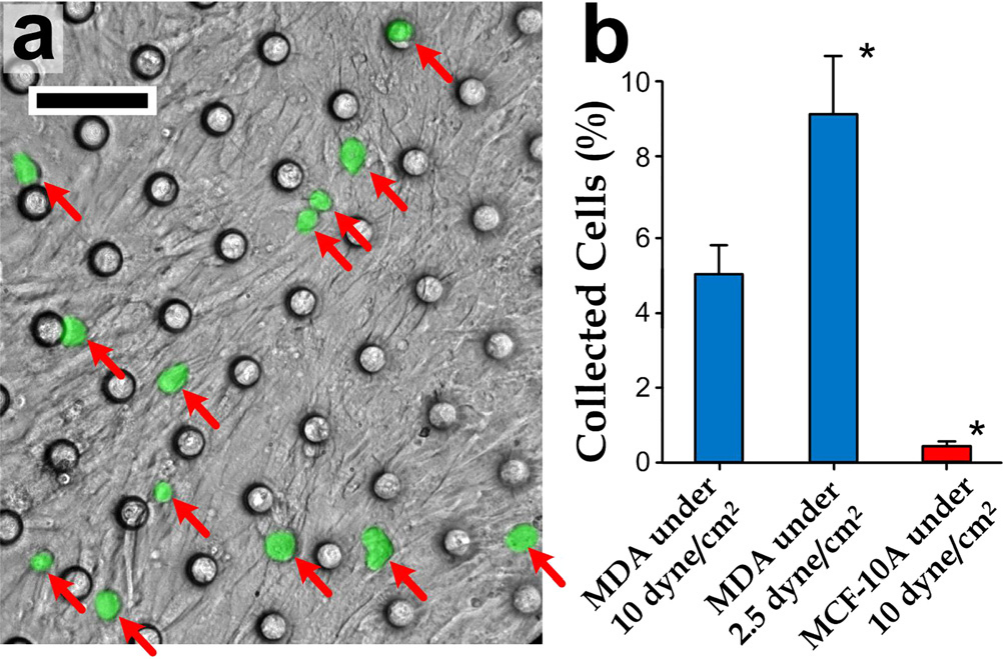
(a) Endothelial layer on a porous membrane with full coverage and further seeded with MDA-MB-231 cells stained with green fluorescence, indicated by arrows (*right*). Scale bar: 100 *μ*m. (b) Population ratios of the collected MDA-MB-231 cells at 10 dyn/cm^2^, MDA-MB-231 cells at 2.5 dyn/cm^2^, and MCF-10 A cells at 10 dyn/cm^2^, obtained from 10 repeated experiments. Each asterisk represents significant difference from the case of MDA-MB-231 at 10 dyn/cm^2^.

Considering the fact that a vast majority of breast cancer-related deaths results from metastases, we have chosen a metastatic breast tumor cell line (MDA-MB-231) to study their transendothelial migration capability and cellular characteristics using the microfluidic porous membrane device. We first seeded and cultured vascular endothelial cells on the membrane (pore diameter: 24 *μ*m) until a cell layer with >90% cell coverage was observed over the membrane region. Next, we prepared the cancer cell sample with a density of ∼2.5 × 10^4^ cells/ml verified by a hemocytometer. A live-cell labeling probe was added in the cell sample to indicate MDA-MB-231 with green fluorescence. We then seeded the cancer cells on the endothelial layer with MDA-MB-231 at a cell coverage of ∼150 cells/mm^2^. A sample micrograph of MDA-MB-231 seeded on an endothelial layer is shown in Fig. 3(a). Afterward, a culture medium with a shear stress of ∼10 dyn/cm^2^ (flow rate: 20 *μ*l/min) was applied along the overhead flow channel (volume above the entire membrane region: 1.6 *μ*l) using a syringe pump. We examined sub-regions of the endothelium and identified the ones with full coverage. For promoting the cell migration, culture media containing 100 ng/ml of the chemokine CXCL12 were then applied along only the cell collection chambers with full coverage of the overhead endothelial cells. After incubating the cells in the device for 15 h,^39,40^ the migrating cells were collected separately by ungating microvalves at the chamber inlets and outlets (only for sub-regions with the full coverage), and flowing trypsin-containing culture media along the collection chambers. Lastly, we counted the collected cells using a flow cytometer, and our results indicate that 5.2% of the MDA-MB-231 cells migrated to the lower collection microchambers (Fig. 3(b)).

To verify the transendothelial migration capability of MDA-MB-231 cells, we repeated the experiments by replacing the cancer cells with a normal breast epithelial cell line (MCF-10A), with results showing that only ∼0.4% of MCF-10A cells could be collected from the microchambers (Fig. 3(b)). Collectively, our results indicate that MDA-MB-231 has a significantly higher transendothelial migration capability than MCF-10A,^41^ agreeing with previously reported observations.^42^

On the other hand, to further examine whether shear stress takes a role in the transendothelial migration, we have repeated the experiments with a lower media flow rate (5 *μ*l/min) inducing a shear stress of 2.5 dyn/cm^2^. Importantly, this platform enables the study on how shear stress affects the migration characteristics, which cannot be offered by the conventional Transwells assays. Our results (Fig. 3(b)) show that significantly more cells (9.1% of MDA-MB-231) could migrate through the endothelial layer compared to the cases with a higher shear stress of ∼10 dyn/cm^2^. One possible explanation is that the shear stress can help maintain the confinement of cell-cell junctions over the endothelial layer.^43,44^ In essence, we have demonstrated that the microfluidic device can be applied to investigate the role of shear stress in the transendothelial migration process of the cancer cells.

### Migration characteristics of the collected migrating cells

**FIG. 4.**
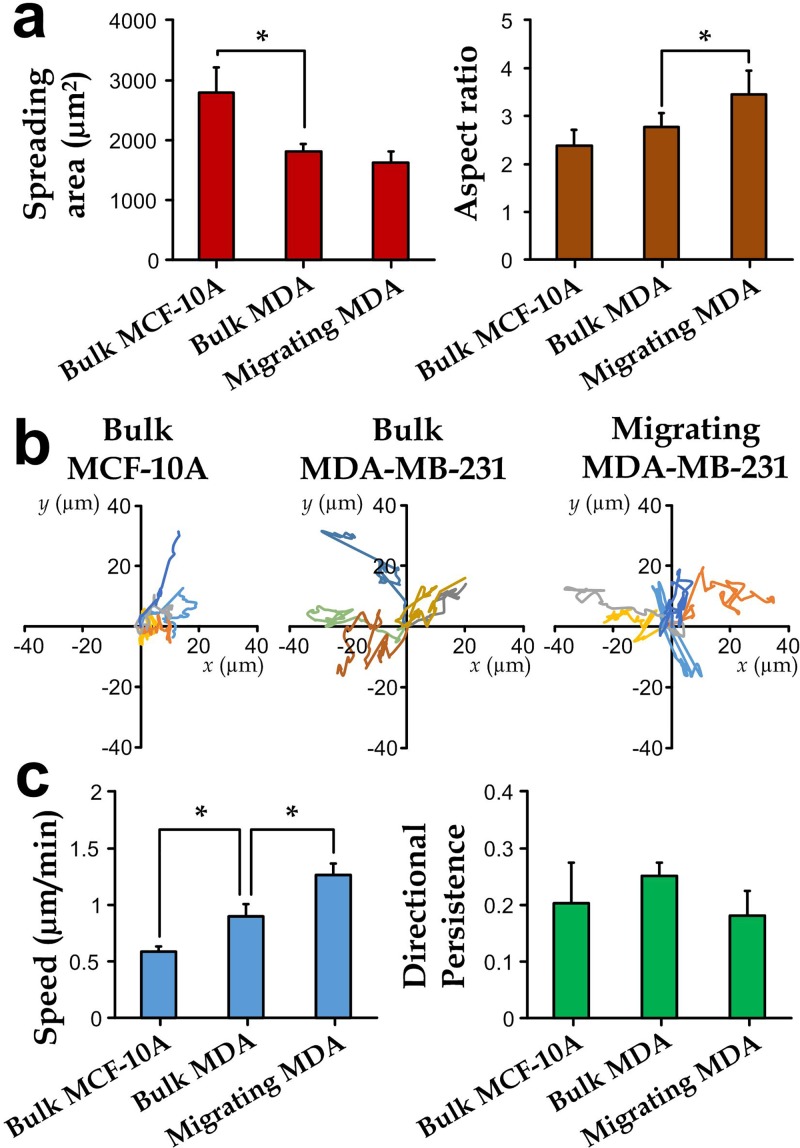
(a) Spreading area and aspect ratio of the cell bodies. (b) Planar migration paths, and (c) migration characteristics (speed and directional persistence) of bulk MCF-10A and bulk and transendothelial migrating MDA-MB-231 cells (N ≥ 10).

It is important to examine whether the transendothelial migrating cells exhibit any morphological and migration ability different from the non-migrating ones. Here, we further applied the microfluidic device with a shear stress of 10 dyn/cm^2^ to extract and analyze the migrating MDA-MB-231 cells for their migration-related properties. The extracted cells were subcultured overnight without the cytokine CXCL12 in order to recover the cell properties before the planar migration analysis. For comparison, we also characterize planar migration for the bulk cultures of MDA-MB-231 and MCF-10A after the 15 h CXCL12 treatment and the overnight recovery. We considered that most cells in the bulk MDA-MB-231 population are the non-migrating cells according to Fig. 3(b). The experimental results (Fig. 4(a)) indicate that the migrating MDA-MB-231 cells exhibited a comparable spreading area and a significantly larger aspect ratio of their bodies than the non-migrating ones. As we have previously shown that the selected membrane pore size (diameter: 24 *μ*m) does not have any selection preference for the cell morphology (Fig. 2(b)), such a higher aspect ratio of the migrating cell bodies should be filtered or induced by the endothelial layer.

We characterized planar cell migration (Fig. 4(b)) using two parameters: speed and persistence. The migration speed was defined as the traveling distance of a cell per minute, whereas the directional persistence was defined as the ratio of the traveling distance and total traveling length over an observation time of 60 min.^45^ Although the faster migration speed of metastatic breast cancer cells over normal breast cells has been previously reported,^46^ it has not yet been demonstrated whether the migration speed is closely related to their transendothelial migration capability. Our results (Fig. 4(c)) further indicate that the faster migration speed of migrating MDA-MB-231 cells should correspond to the transendothelial migration. On the other hand, we observed no significant difference on persistence among the selected cell types.

### Cytoskeletal characteristics of migrating cells

**FIG. 5.**
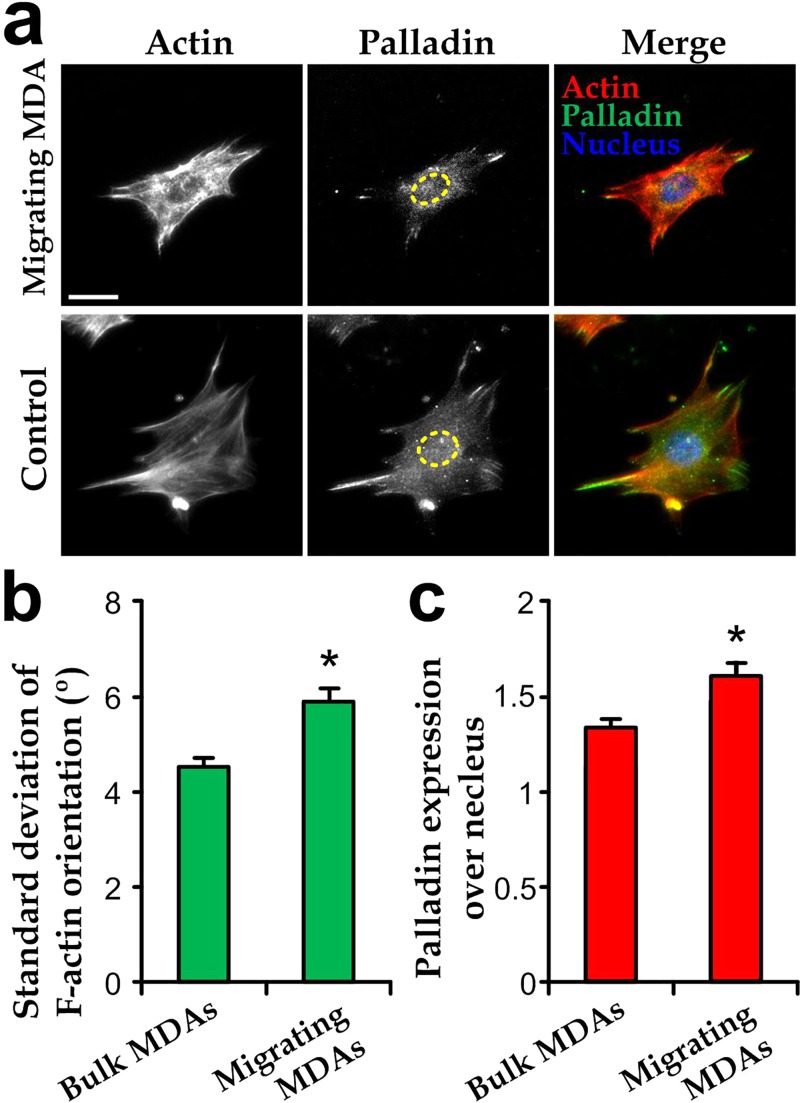
(a) Representative fluorescence images of an extracted migrating MDA-MB-231 cell stained with F-actin, palladin, and nucleus after transendothelial migration and in bulk culture (control). The nucleus regions are highlighted by the dotted lines. Scale bar: 10 *μ*m. (b) Standard deviations of local F-actin orientations over cell bodies. (c) Expression levels of palladin over the nucleus region (N ≥ 20).

We applied the microfluidic transendothelial migration assay (shear stress: 10 dyn/cm^2^) again to the migrating cells, before subculturing them in well plates and quantifying them using conventional cell analysis techniques. As demonstration, we have applied immunofluorescence staining of nucleus, cytoskeletal actin, and palladin on the extracted MDA-MB-231 cells as shown in Fig. 5(a) (*upper*). We also stained and analyzed the MDA-MB-231 cells prepared in bulk culture for comparison (Fig. 5(b), *lower*). It can be observed that the migrating cells exhibited a more random distribution of the filamentous actin, whereas the cells in bulk culture have most actin tended to align with the major cell body direction. We further quantified for local orientations of the actin over cell bodies (as described in Materials and Methods and supplementary material Fig. S1), and the results indicate that the migrating MDA-MB-231 cells indeed have a larger statistical deviation in their local actin orientations.

Our results show that the palladin expression over the nuclear region in migrating cells is higher than that in the non-migrating cells (Fig. 5(c)), suggesting that the nuclear palladin expression may reflect the transendothelial capability of individual cancer cells. In fact, the palladin expression in cancer cells is known to be closely related to cancer metastasis.^47–49^ Although palladin presents in both cytoplasm and nucleus regions, palladin localization in the nucleus^1,34,40^ has been proven to be an indicator for processes such as the F-actin rearrangement and differentiation of cancer-associated cells.^4^ Collectively, the microfluidic device can isolate the migrating cancer cells with a higher specificity in order to further characterize the intracellular cell components closely related to transendothelial migration.

## MATERIALS AND METHODS

### Fabrication

We adopted photolithography of the SU-8 negative photoresist (2025, Microchem, Massachusetts, USA) to fabricate a porous membrane (thickness: 20 *μ*m). Before the process, we coated a molecular layer of (tridecafluoro-1,1,2,2-tetrahydrooctyl)-1 trichlorosilane on a silicon wafer by vaporization, in order to facilitate release of the SU-8 membrane afterward. The SU-8 surface was then coated with 3-aminopropyltriethoxysilane by vapor silanization for 2 h for facilitating bonding in the later steps.^50^

For the device fabrication, we manufactured molds for the replica molding of polydimethylsiloxane (PDMS) as three structural layers: the control layer, and the channel structures above (the flow channel) and underneath (the collection chambers) the porous membrane. The mold for the upper flow channel structures contained a patterned 100 *μ*m thick SU-8 layer on a silicon wafer, and the control layer mold contained a layer of 20 *μ*m of SU-8 on another silicon wafer. Furthermore, we used photolithography to fabricate the mold for the control layer by patterning AZ50XT photoresist (AZ Electronic Materials, Luxembourg, Germany) with a thickness of 60 *μ*m on a silicon wafer. The reflow process was then performed by baking the AZ-silicon mold on a hotplate at 130 °C for 1 min to achieve rounded cross-sections of the microchannels. To facilitate the release after molding for the later process, all the molds were silanized by (tridecafluoro-1, 1, 2, 2-tetrahydrooctyl)-1trichlorosilane vapor overnight.

Afterwards, we fabricated the device mainly based on multilayer soft lithography.^51^ We applied replica molding to obtain a PDMS substrate with a thickness of 4 mm for the upper channel. We applied multilayer soft lithography^34^ to fabricate the stacked PDMS substrate with the collection chamber layer aligned and bonded with the microvalves in the control layer. The flow layer was bonded onto the SU-8 membrane using air plasma (medium level for 1 min; Harrick Plasma, NY, USA) and baked at 70 °C overnight. After peeling off the combined substrate, we treated the other side of SU-8 membrane by 3-aminopropyltriethoxysilane again. We then bonded the combined substrate to the lower chamber layer using the air plasma. To finish the process, we further bonded the entire PDMS substrate to a glass slide using the air plasma again for the physical support.

### Cell culture

Primary human vascular endothelial cells (cat# CC-2519, Lonza) were cultured in an endothelial cell growth medium (EGM-2 BulletKit, Lonza). We only used the cells with a passage number <8 to ensure the cell phenotype. Human MDA-MB-231 breast cancer cells (92020424, Sigma-Aldrich) were cultured in DMEM F12 (cat# D6421, Sigma-Aldrich) supplemented with 10% fetal bovine serum and 1% penicillin. MCF-10A cells (cat# 10317, American Type Culture Collection, VA, USA) were cultured in Dulbecco's Modified Eagle's Medium (DMEM; cat# D5546, Sigma-Aldrich, MO, USA) supplemented with 10% fetal bovine serum (FBS) and 1% penicillin/streptomycin (Sigma-Aldrich). All the cells were cultured in an incubator with a humidified and 5% CO_2_ environment at 37 °C and were passaged once they reached 80%–90% confluence in the culture wells.

### Device preparation and cell seeding

We sterilized the microfabricated device by soaking it in ethanol, ultraviolet exposure and washing all microchannels and microchambers with phosphate buffer saline (PBS). We treated the membrane surface to facilitate cells' attachment and growth by flowing poly-D-lysine (4 *μ*g/cm^2^)^52^ and 50 *μ*g/ml fibronectin along the overhead flow layer with all microvalves in the collection chamber layer closed. We then replaced solutions in all flow regions of the device by fresh culture media.

We seeded endothelial cells with a seeding density of around 8 × 10^4^ cells/cm^2^, by inserting the cells along the overhead channel. After culturing the cells for 4 h, we applied fresh culture media along the overhead channel to remove any unattached cells. We inoculated the endothelial layer by placing the device in a humidified incubator and imposing a continuous medium flow rate of 20 *μ*l/min using a syringe pump (NE300, New Era Pump Systems, NY, USA) overnight. Afterward, we prepared the suspended epithelial cells (MCF-10A or MDA-MB-231 cells) with a density of 2.5 × 10^4^ cells/ml in a mixture of 50% epithelial cell media and 50% endothelial cell media. We injected the cell sample along the overhead flow channel and continued incubation for 4 h such that the cells would be seeded on the endothelial layer; meanwhile, CXCL12 in the culture medium was flowing along the selected collection chambers fully covered by an endothelial region.

### Cell viability assay

We applied the LIVE/DEAD Viability/Cytotoxicity Kit (cat# L3244, Thermo Fisher Scientific) to examine transmigrating cell viability. Cells were first collected from the selected microchambers underneath the fabricated membrane by flowing culture media along the microchambers. We then seeded cells on a sterile glass coverslip in a 35 mm disposable Petri dish for 2 h, followed by adding Calcein AM (2 *μ*M) and EthD-1 (4 *μ*M) (Sigma-Aldrich) in the culture medium. After incubating for 30–45 min at room temperature, fluorescence images were captured using an inverted fluorescence microscope (TE300, Nikon, Tokyo, Japan).

### Flow cytometry

Before seeding breast cells onto an endothelium in the microfluidic device, we utilized cell tracker (CellTracker™ Green CMFDA Dye, Thermo Fisher Scientific, Grand Island, NY) at a concentration of 1 *μ*M in serum-free media to stain the cells with fluorescence signals. After centrifuging and removing the supernatant, fresh culture media without the cell tracker were added back in the cell sample until a target cell density was obtained. After the cells migrated through the endothelium during the experiment, we gathered the migrating cells from the collection chambers. We then counted the migrating cells using a flow cytometer (Accuri C6 Plus, BD Biosciences, CA, USA).

### Quantification of planar migration characteristics

The collected cells were seeded onto a coverslip placed in a Petri dish and cultured for 2 h in a conventional incubator for cell attachment and spreading. Afterward, the Petri dish was then incubated with a bright-field microscope (TE300, Nikon, Tokyo, Japan) equipped with controls of temperature (37 °C), humidity (100%), and 5% CO_2_ in air for another 2 h; meanwhile, the cell migration was monitored by taking time-lapsed micrographs every minute using a scientific complementary metal-oxide-semiconductor (sCMOS) camera (Zyla 4.2, Andor, Belfast, UK) installed with the microscope. Later, the time-lapsed images were processed with ImageJ (National Institutes of Health, MD, USA) to determine cell centroid positions at different time points. We characterized the planar migration behaviors with (1) the migration speed defined as the cell traveling length per minute and (2) migration persistence defined as the ratio of migration distance and traveling length over the last 1 h of monitoring.

### Quantification of actin orientation

We adopted the procedures previously described by Yushigi *et al*.^53^ to quantify the subcellular orientations of stress fibers. Briefly, the edge detection process was applied on the actin-stained fluorescence images of a cell (supplementary material, Fig. S1(a)). Two Sobel filters were then applied to determine the horizontal and vertical gradients of the local image intensity (Fig. S1(b)). The stress fiber orientation at every pixel was calculated by the arctangent of the ratio between the vertical and horizontal gradients (Fig. S1(c)). We considered the standard deviation of the subcellular fiber orientations (Fig. S1(d)) to reflect the alignment of stress fibers: a smaller deviation represents more consistent alignment of the stress fibers, and vice versa.

### Quantification of palladin expression in the nucleus region

We analyzed the palladin expression over a nucleus region by first segmenting the nuclear region based on the 4′,6-diamidino-2-phenylindole (DAPI) fluorescence, followed by calculating the average image intensities in the corresponding nucleus region in the palladin-stained images. The normalized palladin expression in the nucleus region was then calculated by the ratio between the palladin expression in the nucleus region and average palladin intensity over the cell body.

### Immunofluorescence staining

Cells extracted from the selected collection chambers of the device were subcultured on coverslips without CXCL12 overnight. They were fixed by 4% (w/v) paraformaldehyde (PFA, Electron Microscopy Science, Hatfield, PA) for 30 min, followed by washing them thoroughly with filtered PBS for 3 times and then permeabilization with 0.3% Triton X-100 in PBS for 5 min. After rinsing with PBS, the cells were immersed in a blocking solution (10% goat serum in PBS; Invitrogen) for 1 h to eliminate any non-specific binding. We incubated the samples with a diluted rabbit anti-palladin primary antibody (Sigma-Aldrich, St. Louis, MO) in the blocking solution with a concentration of 5 *μ*g/ml for 1 h at room temperature, followed by staining the sample with Alexa Fluor 488 conjugated goat anti-rabbit IgG secondary antibody (Invitrogen) for another 1 h. After washing with PBS for 3 times, Alexa Fluor 555 conjugated palladin (Invitrogen) and 4′,6-diamidino-2-phenylindole (DAPI, Invitrogen) were further applied to label F-actin and nucleus.

### Statistics

Error bars in plots are standard errors. We compared two groups of data for any significant difference using Student's two-tailed, unpaired T-test to obtain the *p*-values. An asterisk represents a significant difference between two data groups (*p* < 0.05).

## CONCLUSION

We report a microfluidic device to isolate and investigate the transendothelial migrating cancer cells, with a unique advantage of isolating cells migrated through a fully covering endothelium for high collection specificity. We have optimized the pore diameter of the microstructured supporting membrane to be 24 *μ*m in order to facilitate the endothelium formation and exclude cell changes caused by physical constriction of the pores. This device can isolate migrating cancer cells and reveal the cell characteristics related to the transendothelial migration capability of a malignant human breast cancer cells (MDA-MB-231). In essence, we have applied this device to isolate the migrating MDA-MB-213 cells and reveal their properties of a higher body aspect ratio, faster planar migration, less consistent stress fiber alignment, and a higher nuclear palladin expression, compared to the non-migrating ones. Notably, our platform can be configured with an optimized pore size on the microfabricated membrane to investigate the transmigration capability of other cancer cells such as prostate cancer cells and brain cancer cells. The platform has a high potential to flow with biofluids such as the human serum or the whole blood for more realistic biophysical conditions, with improvements such as a larger membrane region. Future applications of this integrated microfluidic transendothelial migration assay can include investigation for the biochemical factors, e.g., chemokines, inflammatory molecules, and pharmacological drugs, affecting cancer cells on their transendothelial migration as well as invasion (intravasation and extravasation) characteristics for more effective cancer research and drug development.

## SUPPLEMENTARY MATERIAL

See supplementary material for an example of the actin orientation quantification in Fig. S1.
